# Doublethink and scale mismatch polarize policies for an invasive tree

**DOI:** 10.1371/journal.pone.0189733

**Published:** 2018-03-07

**Authors:** Caleb P. Roberts, Daniel R. Uden, Craig R. Allen, Dirac Twidwell

**Affiliations:** 1 Department of Agronomy & Horticulture, University of Nebraska, Lincoln, NE, United States of America; 2 Nebraska Cooperative Fish and Wildlife Research Unit, School of Natural Resources, University of Nebraska, Lincoln, NE, United States of America; 3 U.S. Geological Survey, Nebraska Cooperative Fish and Wildlife Research Unit, School of Natural Resources, University of Nebraska, Lincoln, NE, United States of America; USDA-ARS Fort Keogh Livestock and Range Research Laboratory, UNITED STATES

## Abstract

Mismatches between invasive species management policies and ecological knowledge can lead to profound societal consequences. For this reason, natural resource agencies have adopted the scientifically-based density-impact invasive species curve to guide invasive species management. We use the density-impact model to evaluate how well management policies for a native invader (*Juniperus virginiana*) match scientific guidelines. *Juniperus virginiana* invasion is causing a sub-continental regime shift from grasslands to woodlands in central North America, and its impacts span collapses in endemic diversity, heightened wildfire risk, and crashes in grazing land profitability. We (1) use land cover data to identify the stage of *Juniperus virginiana* invasion for three ecoregions within Nebraska, USA, (2) determine the range of invasion stages at individual land parcel extents within each ecoregion based on the density-impact model, and (3) determine policy alignment and mismatches relative to the density-impact model in order to assess their potential to meet sustainability targets and avoid societal impacts as *Juniperus virginiana* abundance increases. We found that nearly all policies evidenced doublethink and policy-ecology mismatches, for instance, promoting spread of *Juniperus virginiana* regardless of invasion stage while simultaneously managing it as a native invader in the same ecoregion. Like other invasive species, theory and literature for this native invader indicate that the consequences of invasion are unlikely to be prevented if policies fail to prioritize management at incipient invasion stages. Theory suggests a more realistic approach would be to align policy with the stage of invasion at local and ecoregion management scales. There is a need for scientists, policy makers, and ecosystem managers to move past ideologies governing native versus non-native invader classification and toward a framework that accounts for the uniqueness of native species invasions, their anthropogenic drivers, and their impacts on ecosystem services.

## Introduction

To avoid ecological and economic consequences from invasive species [1, 2], natural resources agencies must make and follow proactive, scientifically-supported invasive species management policies [3, 4]. Mismatches between policy and ecology can lead natural resource agencies toward a “doublethink” mentality (where contradictory thoughts exist without acknowledged cognitive dissonance) that produces policies that simultaneously promote and control invasive species [5, 6]. This doublethink mentality manifests from the need to respond to opposite demands of diverse citizenry, misunderstanding scales of invasion, and “nebulous” concepts such as native invaders [7–11]. The actions and behaviors resulting from doublethink misalign invasive species management policies with basic ecological invasion theory. This mismatch eliminates the potential for management tactics to meet goals meant to prevent [12], eradicate [13], or simply control [14] invasions, thereby increasing the likelihood for sharp declines in ecosystem services and regime shifts to hysteretic, undesirable alternative states.

**Fig 1:**
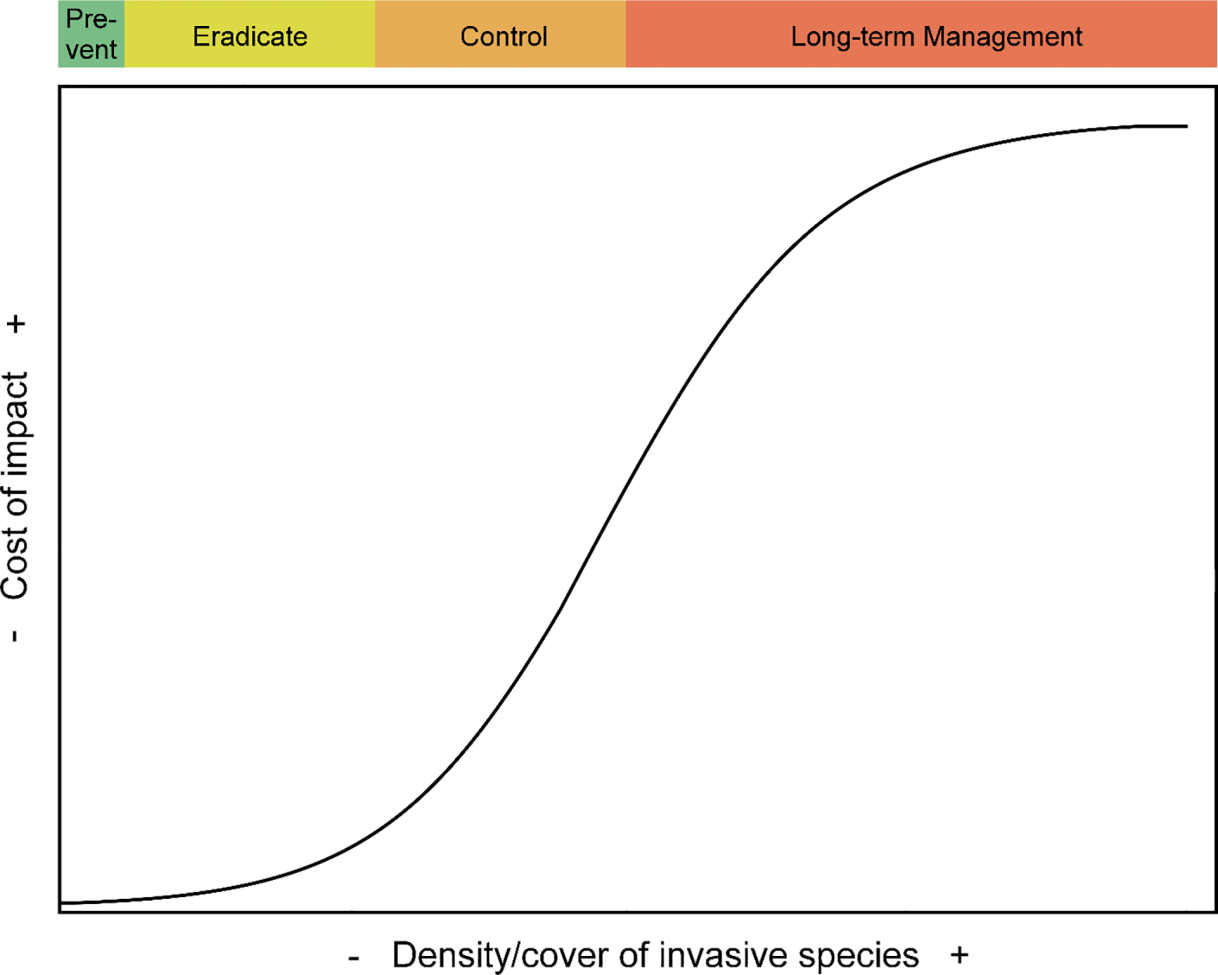
Type II density-impact invasion curve adapted from Yokomizo et al. (2009). Depicts the sigmoidal relationship between an invasive species’ density or area covered and the cost or impact to socio-ecological systems. The colored bar above the curve shows preferable management strategies for the four stages of invasion based on feasibility, likelihood of success, and cost-effectiveness.

Natural resource agencies have adopted the scientifically-derived “density-impact invasive species curve” model to guide management actions, prioritize investments, and prevent policy-ecology mismatches [15]. The density-impact invasive species curve (hereafter the “density-impact model”) provides theoretical insight into the economic impacts of invasive species as they increase in abundance and density over time (Fig 1). The model has been used as the basis for understanding the feasibility and effectiveness of management strategies at different stages of the invasion process, and it can help identify mismatches in policy where doublethink poses large risks to economic assets. Misunderstanding the density-impact model results in doublethink management that under-invests (lags behind) at the early stages of invasion and then over-invests (tries to catch up) at later stages [12–16].

Invasive species policy has been slow to adapt to a non-stationary and rapidly changing world [17]. Species that did not pose risks in the past but which are now invading and responsible for major socio-economic losses due to their impacts to other resources are particularly likely to escape policies built around assumptions of equilibrium [4, 5]. A key uncertainty surrounds native invaders, which are now known to have impacts that rival non-native invaders but which also have traits that make them valuable in specific contexts [7, 11]. It is unclear how disparate existing policies are for native invaders that are known to have strong density-related impacts.

We use the density-impact model to identify the degree of mismatch between science and policy for a native invader in central North America, eastern redcedar (*Juniperus virginiana*). Scientists and natural resource professionals have reached consensus on the invasive potential and sharp social, economic, and ecological impacts of this native tree as its density increases [18–20]. Our policy assessment stems from collaborations with USA federal and state agencies seeking to better conserve temperate grasslands at the front-line of juniper invasions in the Great Plains. In this paper, we (1) use land cover data to identify the stage of eastern redcedar invasion for three ecoregions within the State of Nebraska, USA, (2) determine the range of invasion stages at individual land parcel extents within each ecoregion based on the density-impact model, and (3) determine policy alignment and mismatches relative to the density-impact model in order to assess their potential to meet sustainability targets and avoid societal impacts as eastern redcedar abundance increases.

## Methods

### Focal species

Eastern redcedar is a dioecious, non-resprouting conifer that has the broadest range of any conifer in North America [21]. Due to human fire suppression and intentional planting, eastern redcedar has become a native invader in the Great Plains of the USA—expanding its geographic range at an exponential rate [22]—and has initiated biome-level regime shifts from grasslands to woodlands [21, 23, 24]. Historically, frequent human burning of Great Plains landscapes constrained eastern redcedar’s geographic distribution to isolated locations (e.g., lowlands and rough topographies) [9, 25, 26]. In the absence of fire, eastern redcedar has no known environmental filter except open water and wetland sites [21, 27], meaning only dispersal time (lag) and abiotic factors that influence rate of invasion (e.g., soil type, precipitation) mediate a given site’s vulnerability to invasion [26, 28].

Eastern redcedar invasion incurs sharp social and economic losses due to reduction in profitability of grazing lands [23, 29, 30], loss of public school funding [20, 31], heightened wildfire risk [20], and reductions in the diversity and abundance of endemic taxa, in some cases causing local extinctions [32–34]. More than 50 years of study on *Juniperus* species in the Great Plains have provided detailed knowledge of invasion pattern, spread rates, and sensitivity to management tactics [30, 35, 36], but the high cost of mechanical removal and policy-driven fire suppression inhibit management at spatial extents greater than individual land parcels [31].

Eastern redcedar shares basic mechanisms of spread with non-native invaders (e.g., removal of environmental constraints induces spread, temporal lags in invasion, density-impact relationship) [22–24, 28, 30] and incurs costs that rival other non-native invaders in North America [18, 31]. Despite these similarities with non-native invaders, the uniqueness of native invaders such as eastern redcedar can lead to doublethink in management actions [5, 7, 11]. For instance, because prevention and eradication must be scale-dependent strategies for native invaders (i.e., eradicating a native invader from many adjacent land parcels may be desirable, but not from its entire historic range), doublethink can occur if policies prohibit or fail to incentivize prevention or eradication at any scale [37]. This can lead to waiting to initialize management until the exponential growth phase (the control stage), or in some instances, density-impacts may have already passed acceptable thresholds, making only long-term mitigation and resource protection feasible [1, 2, 14, 15, 38]. Additionally, native invaders, such as eastern redcedar, can be profitable in certain circumstances (e.g., selling seedlings grown in government-funded nurseries) [7], but also incur steep social, economic, and ecological costs that natural resources agencies must manage [18].

In this paper, we evaluated if policy for managing eastern redcedar invasion matches the density-impact model in Nebraska. Although many regions within the USA’s southern plains have already reached the carrying capacity/local control stage of invasion, northern plains states such as Nebraska are on the “front line” of eastern redcedar invasion and have not yet experienced regime shifts at broader spatial extents [9, 29]. Natural resource agencies in front line states have the opportunity to halt ecological regime shifts and economic losses by developing and implementing policy that matches ecological knowledge [24]. In 2014, the Nebraska state Conservation Roundtable identified eastern redcedar as one of the greatest threats to conservation in the state [39]. Since then, momentum for matching policy and ecology for eastern redcedar management has grown, with the Nebraska Invasive Species Council affirming eastern redcedar as a native invader and multiple local stakeholders and working groups calling for more proactive and ecologically-supported policy and management actions [19, 40, 41].

### Study site

**Fig 2:**
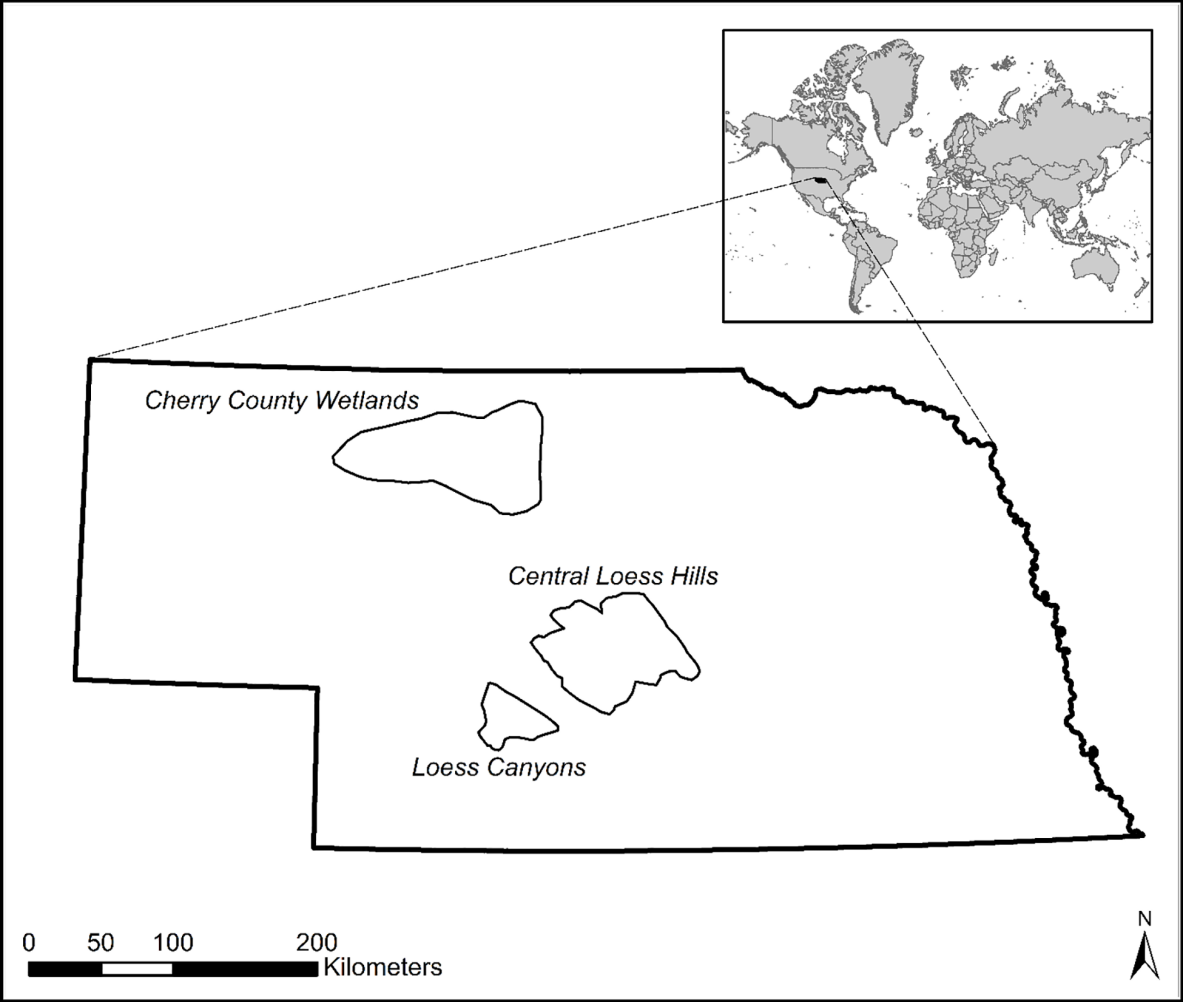
Study site. Locations of three ecoregions that are a priority for grassland conservation, according to the Nebraska Legacy Project, and which form the basis for assessing the degree of concordance between policy and science in the management of the native invader eastern redcedar (*Juniperus virginiana*) in Nebraska, USA.

In Nebraska, the Nebraska Natural Legacy Project [42] serves as the State Wildlife Action Plan. The overarching objectives of the Nebraska Natural Legacy Project are to conserve the flora, fauna, and natural habitats of the state. To achieve these objectives, management actions are focused in 39 ecoregions termed “biologically unique landscapes” (hereafter referred to simply as “ecoregions”) across the state, which collectively offer opportunities for conserving the full array of the state’s biodiversity. We chose three ecoregions within Nebraska that were historically grass-dominated systems and that have been invaded by eastern redcedar to varying degrees: the Cherry County Wetlands, the Central Loess Hills, and the Loess Canyons (Fig 2) [43]. We also selected these ecoregions because the Nebraska Invasive Species Council [19] and stakeholders have identified eastern redcedar as a potential “resource concern” (i.e., threatening economic, ecological, and social resources) to federal and state agencies in these ecoregions. We obtained elevation data for our study sites from 30 meter resolution digital elevation model imagery downloaded from the website of the Nebraska Department of Natural Resources [44]. We obtained precipitation data for our study sites by summing mean monthly precipitation values from one kilometer resolution mean monthly precipitation raster data over a 50-year time period (1950–2000) in each ecoregion [45].

#### Cherry County Wetlands

The Cherry County Wetlands ecoregion is located in the northern portion of the Sandhills in Cherry County, Nebraska [42]. Numerous lakes, wet meadows, marshes, and fens are situated in valleys between sand dunes covered in relatively unbroken Sandhills mixed-grass prairie. Agricultural land use consists primarily of haying and cattle grazing, although some row crop production is supported by center-pivot irrigation in river valleys. Elevation ranges from 778 to 1,227 meters above sea level. Mean average annual precipitation between the years 1950 and 2000 ranged from 461 to 570 millimeters (mm).

#### Central Loess Hills

The Central Loess Hills ecoregion is located in central Nebraska and consists of rolling to steep hills dissected by the Middle Loup and North Loup River Valleys [42]. Hilly upland areas were traditionally reserved for grazing, but rowcrop production is now moving beyond the flat river valleys and into farmable upland areas. Playa wetlands can be found in relatively high densities in the northwestern portion of the landscape. Although grassland and cropland are the dominant land cover classes, the spread of eastern redcedar is increasing the proportion of the landscape in woodland/forest [42]. Elevation ranges from 612 to 951 meters above sea level. Mean average annual precipitation ranged from 555 to 647 mm between the years 1950 and 2000.

#### Loess Canyons

The Loess Canyons ecoregion is situated south of the Platte River in portions of Lincoln, Dawson, and Frontier Counties of Nebraska [42]. Steep loess hills and canyons are characteristic landscape elements, with row crop fields being interspersed amidst blocks of mixed-grass prairie. Several historical accounts indicate the presence of dense stands of eastern redcedar in local canyons along the Platte River in the mid-nineteenth century, but not in upland areas beyond the river [46, 47]. In recent decades, the spread of eastern redcedar trees into upland prairies has become an economic and ecological concern, and removal projects by private landowners and conservation organizations are underway [42]. Elevation ranges from 781 to 989 meters above sea level. Mean average annual precipitation ranged from 510 to 563 millimeters between the years 1950 and 2000.

### Data collection

#### Policy identification

**Fig 3:**
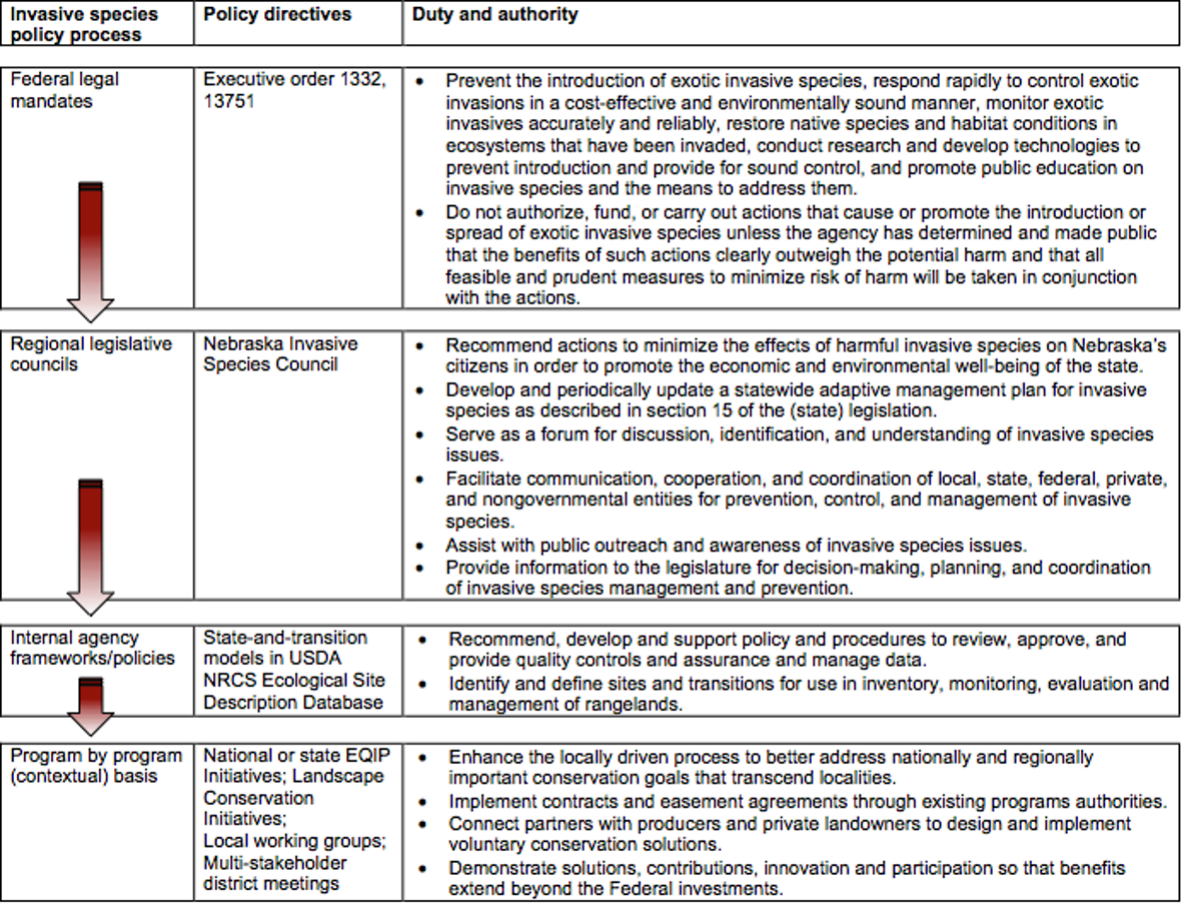
Outline of the hierarchical invasive species management policy creation process for USA natural resources agencies. Arrows indicate the direction of hierarchical control, with federal legal mandates being the highest level. Columns represent the policy creation process (first column), examples of groups that create, enforce, and implement the policies (second column), and the policy-directed duties of the groups at each level (third column).

Financial incentive programs and technical guidance are derived from a hierarchical set of policies used to prioritize the identification and management of invasive species in the USA (Fig 3). This general process was identified as part of our involvement in conservation partnerships with diverse representations spanning public, private, and academic sectors. A key difference between policies for native and non-native invaders is that native invaders are not included in policy directives that establish federal mandates for invasive species management (e.g. Executive orders, Fig 3). Native invaders can only be captured within regional-to-local policy directives.

For the purposes of this assessment, we worked with natural resource agencies to identify specific policies that have become institutionalized in the behaviors and practices of federal and state natural resource agencies. We focused on those policies that most affected decision-making and conservation investments on private lands. Nebraska, like other states throughout the Great Plains, is almost entirely owned and managed by private landowners (97% private land ownership), so we focused less on internal agency policies that were more relevant to public land management. For purposes of transparency, we include our involvement and interactions for each relevant level of the hierarchical policy process.

The Nebraska Invasive Species Council and Nebraska Conservation Roundtable are regional legislative councils that serve as government agency platforms for discussions on eastern redcedar policies. In 2014, the Nebraska Conservation Roundtable crafted a white paper listing eastern redcedar as one of the greatest conservation threats in Nebraska [39]. In 2017, the Nebraska Invasive Species Advisory Council listed eastern redcedar as a ‘problematic’ invader because of scientifically-established information on economic and environmental declines associated with eastern redcedar invasions [19]. This designation overcame traditional tendencies to equate invasive with non-native and to critique the potential efficacy of programs at lower levels of the hierarchy (Fig 3).

With this in mind, we conducted an in-depth search of publicly-available policy documents to go alongside previous assessments of internal agency management frameworks that govern program implementation and technical guidance across millions of acres of private land in the USA [48]. Our in-depth review can be thought of as a *Program by Program* compilation of policy-relevant documents. We reviewed cost-share/financial incentive programs, management plans, official memos, official protocols, or published technical guides for private landowners that related to eastern redcedar management in grasslands, forestry, or agriculture. This resulted in diverse policy representation spanning examples from multiple state and federal groups. We also engaged representatives from private citizen-led organizations in the region, such as the Sandhills Task Force, Loess Canyon Rangeland Alliance, and Nebraska Prescribed Fire Council, to identify landowner perceptions about relevant policies most affecting them. Specific policies are referenced in our policy assessment.

#### Land cover

We used 30 m resolution land cover data for the Nebraska which was provided by the Rainwater Basin Joint Venture (RWBJV) [49]. This land cover dataset was created in 2010 by integrating 12 existing land cover datasets (e.g., the Landsat-derived National Land Cover Dataset, the Natural Agriculture Statistics Service land cover data, and the National Wetlands Inventory), in which classifications of layers higher in the stack superseded those of lower layers [Bishop et al. 2011]. We chose the RWBJV dataset because it represents the most current Nebraska land cover dataset containing eastern redcedar as an explicit cover class. The resulting land cover was classified and hierarchically organized into divisions (i.e., level 1), types (i.e., level 2), associations (i.e., level 3), and conditions (i.e., level 4). Full details about the RWBJV dataset can be found in the RWBJV’s report [49].

The RWBJV dataset includes a specific eastern redcedar cover class, which we used to estimate percent cover of eastern redcedar in each ecoregion and the range of cover across individual land parcels. Within the hierarchical classification of the RWBJV dataset, the Eastern Redcedar cover class is nested within the Upland Forest/Woodland Association, which is nested within the Forests/Woodlands type, which is nested within the Terrestrial Division. In an informal evaluation, Bishop et al. (2011) found classification accuracy in the dataset to be ~95% for broad landscape categories (e.g., cropland, grassland, and woodland). Because the 30 m resolution of the RWBJV dataset may not detect low densities of eastern redcedar (incipient invasions) and a large proportion of the higher level classification of upland forest/woodland is likely eastern redcedar (but was not classified as eastern redcedar due to lower certainty), we expect eastern redcedar’s cover across ecoregions to be somewhat under-represented [49]. Because eastern redcedar is actively removed from agricultural croplands and cannot invade extremely mesic sites (e.g., open water, riverine systems, and wetlands), we excluded these classes from the analysis.

We estimated eastern redcedar invasion stage by calculating percent coverage of eastern redcedar from RWBJV land cover data at the extent of each ecoregion. We selected ecoregions because many management strategies and priorities in Nebraska are directly or indirectly associated with the Natural Legacy Plan, which focuses on ecoregion-level planning and action across the entire state [42]. We classified invasion stages according to the density-impact model, which establishes four invasive species management strategy stages based on sections of the sigmoidal curve and the range of invasive species percent cover/densities over which each strategy is most cost-effective. We derived specific percent cover cutoffs for each invasion stage classification from literature that quantified eastern redcedar’s sigmoidal density/cover-economic impacts (e.g., the livestock industry) [18] and grassland taxa biodiversity [34] relationship. The relationship between density-impact and population growth pattern has been established as sigmoidal for *Juniperus viriginiana*, although there is uncertainty in exact locations of density-impact curve inflection points due to site differences (soils, precipitation) and the case-study nature of past published studies [18, 30, 34]. Management and policy are tied to stages of invasive species’ sigmoidal population growth and impacts patterns, with prevention being the strategy when an invasive species is absent or rare, eradication being the strategy during incipient invasion up until the beginning of rapid increase in impact/population just before peak growth rate on the sigmoidal curve, control being the strategy during the peak impact/population growth rate, and long-term management being the strategy just before the impact/population growth rate declines and becomes asymptotic [15]. The point at which impact approaches its maximum in the eastern redcedar density-impact relationship ranges between 60 and 90% cover due to site-dependent uncertainty (e.g., differences in soil type, precipitation) [18, 21], but because invasion ecology theory establishes the benefits of early and quick management actions, we established our invasion stage cutoff based on the lowest (60% cover) estimate [15, 16]. Accordingly, we classified 0–<1% cover as the prevention stage, 1–10% cover as the eradication stage, 11–59% cover as the control stage, and >60% cover as the long-term management stage.

We then quantified the range of eastern redcedar cover in individual land parcels within each ecoregion. We received agency data to define the scale at which financial incentives programs are being operationalized on private lands and, thus, the appropriate scale to use as the basis for our policy assessment. Assessment of agency data showed eastern redcedar management policies are on average operationalized at 50-acre (approximately 20.2 ha) land parcels in Nebraska [31]. For each ecoregion, we searched for the presence or absence of 20.2 ha cells (approximately 450 m x 450 m, or 225 pixels at 30 m resolution) that fell within each of the four invasion stages we established; that is, we searched for at least one 20.2 ha cell in which percent eastern redcedar percent cover corresponded to the prevention stage (0–<1% cover), one cell for the eradication stage (1–10% cover), etc.

### Evaluating policy-ecology agreement

We used policy and land cover data to assess the level of agreement between the specific eastern redcedar management policies of agencies and the general management recommendations derived from the density-impact model [15]. Our policy assessment focused on two functional scales: (1) the extent of individual ecoregions, and (2) the average spatial extent used for policy delivery. Both spatial scales are currently used to make policy decisions and to provide technical guidance, even though related policies are not spatially-explicit.

## Results

### Ecoregion-level abundance

**Fig 4:**
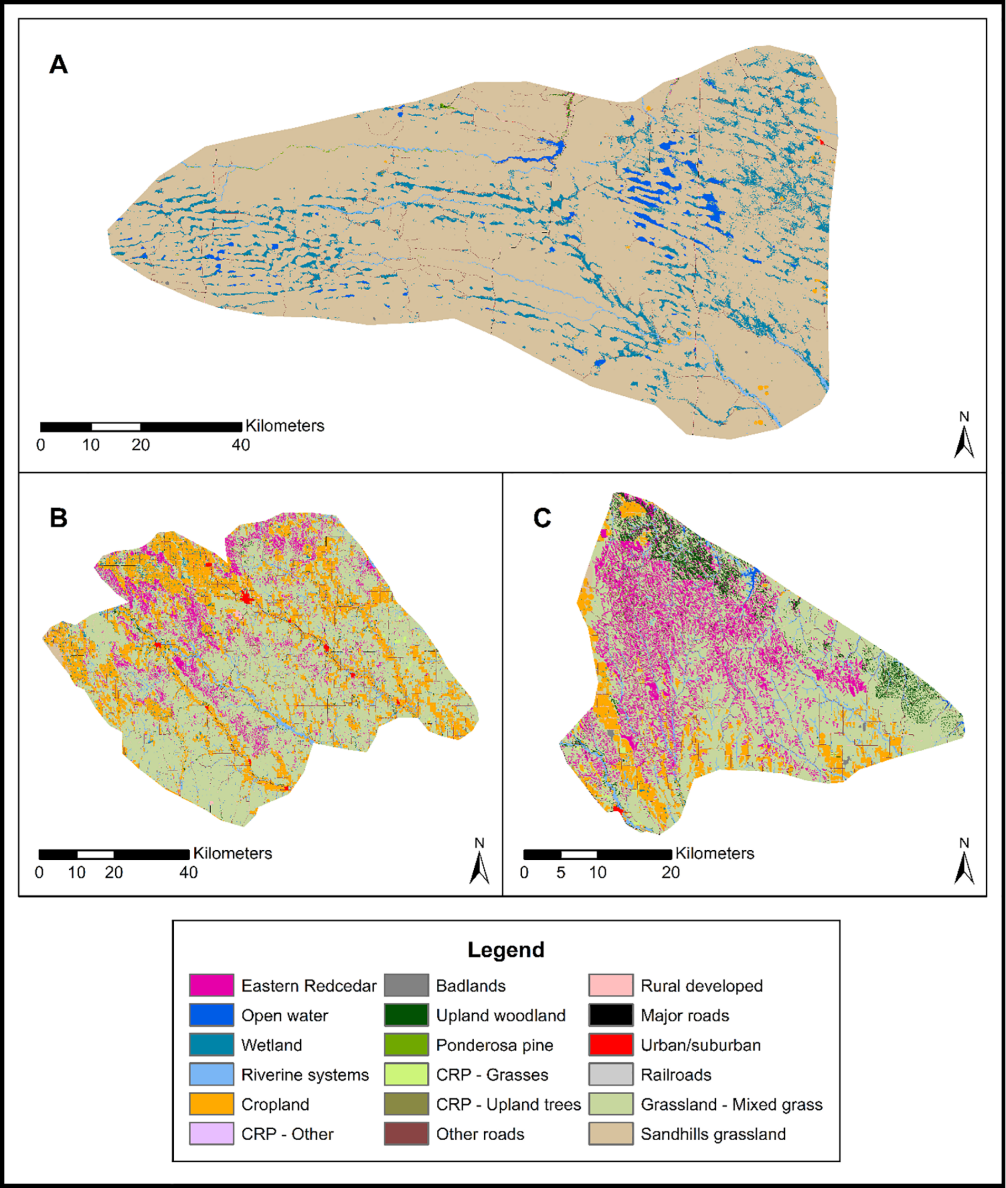
Land cover classifications for the three ecoregions used in our assessment. A: Cherry County Wetlands, B: Central Loess Hills, C: Loess Canyons. Dataset used with permission from the Rainwater Basin Joint Venture.

Land cover data identified the stage of eastern redcedar invasion for three historically grass-dominated ecoregions within Nebraska (Table 1). Estimates of juniper woodland ranged from 19.66% in the Loess Canyons to 0.02% in the Cherry County Wetlands (Fig 4). Juniper woodland occurred on 9.54% of the Central Loess Hills (Fig 4).

**Table 1:** Percent land cover estimates for three ecoregions in Nebraska, USA. Columns indicate ecoregion name, the total land area within each ecoregion, the subset of land within ecoregions susceptible to eastern redcedar (ERC) invasion, the total land area classified as eastern redcedar, and the percent of susceptible land (i.e., cropland, riverine systems, wetland, and open water classes were not susceptible and were therefore removed from the analysis) covered by eastern redcedar. Land cover was estimated in 2010 and is derived from the Rainwater Basin Joint Venture 30 m remotely-sensed land cover dataset.

Ecoregion	Total Area (ha)	Area Susceptible to ERC (ha)	ERC Cover Area (ha)	Percent ERC Cover
Cherry County Wetlands	709,202.07	627,533.73	140.49	0.02%
Central Loess Hills	567,620.19	389,500.02	37,168.29	9.54%
Loess Canyons	136,765.53	112,993.74	22,214.79	19.66%

### Individual land parcels (20-ha)

**Fig 5:**
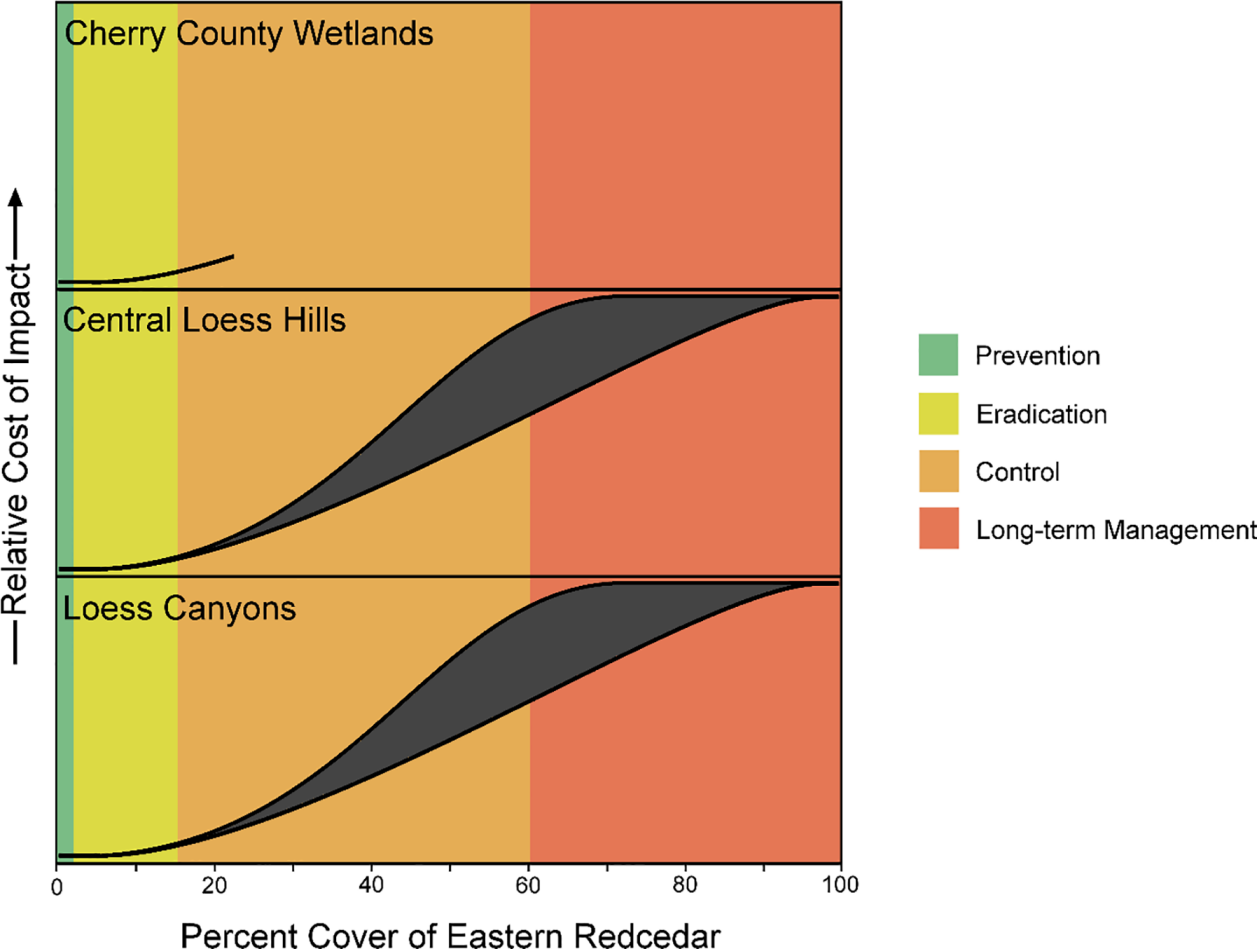
The relative impact costs of eastern redcedar on individual land parcels in each ecoregion. Shown here is the range of impacts related to abundance (not the relative distribution of land with eastern redcedar cover within each ecoregion). Sigmoidal line lengths depict the range of eastern redcedar density/cover located in at least one 50 acre (20.2 ha; 450 m x 450 meter) cell in each ecoregion in 2010, relative to eastern redcedar’s known sigmoidal density-impact relationship.

**Fig 6:**
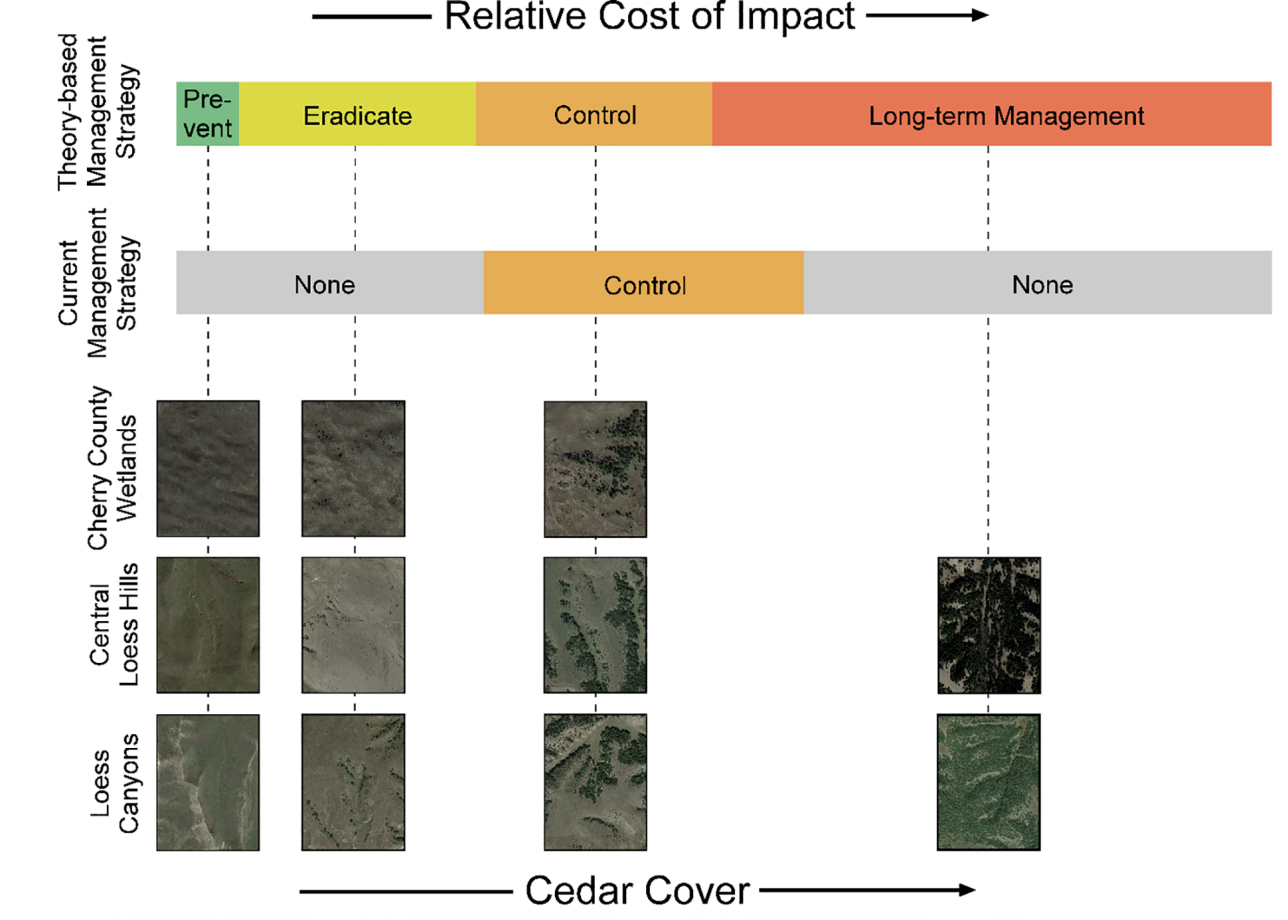
Mismatch between the science and policy of managing eastern redcedar invasions in Nebraska, USA. Aerial photographs show examples of each eastern redcedar invasion stage found in 20.2 ha parcels of land from each ecoregion. Photographs are arranged under the “theory-based management strategy” that would maximize the returns based on the density-impact model as well as under the “current management strategy” implemented in natural resources agency policies. As shown here, control of eastern redcedar only occurs during periods of exponential growth. Policies promote spread during incipient invasions and ignore opportunities for restoration following a regime shift.

Data on individual land parcels within each ecoregion showed that the minimum and maximum percent cover of eastern redcedar invasion in the Cherry County Wetlands was 0%–22.67% cover, whereas invasion spanned the full range of cover (0–100% cover) in the Central Loess Hills and Loess Canyons ecoregions (Fig 5). According to the density-impact model adapted to the known density-impact relationship of eastern redcedar [18], individual land parcels in the Cherry County Wetlands spanned the prevention, eradication, and control invasion stages, and land parcels in the Central Loess Hills and Loess Canyons ranged from prevention to long-term management stages (Figs 5 and 6).

### Policy assessment

Our policy assessment revealed that existing policies fail to align with theoretical expectations of the density-impact model for eastern redcedar. At the extent of an ecoregion, there is no evidence for prevention, eradication, or long-term management in policy (Table 2). Instead, policy promotes invasion (Table 2). Planting occurs in all ecoregions studied here. Statewide, natural resource agencies distributed on average 850,000 eastern redcedar seedlings per year from 1925–2001 [7], shifting to approximately 310,000 seedlings per year from 2001 to the present [50]. Moreover, our study confirms that current practices and programs align primarily with levels of invasion that require controls to be implemented (Table 2). The controls are put into practice on a *Program by Program* basis following the identification of eastern redcedar as a resource concern (Fig 2). Controls are then authorized via policy to attempt to freeze the system in the desired range of percent cover. If these controls are not effective, or if they are not implemented prior to reaching a more advanced stage of invasion, then few directives are in place currently to implement long-term management at the extent of an entire ecoregion (Table 2).

**Table 2:** Examples of agency policies and programs used to manage eastern redcedar and how they align with the scientific basis for invasive species management based on the density-impact invasion curve. Asterisks denote agency programs with scientific support.

Invasion stage	Degree of policy alignment with scientific recommendations	Policy description	Examples of agency programs
Prevention	Strongly misaligned	Spread is encouraged and facilitated. Preventative actions are not evident.	• Nurseries grow and distribute seedlings^[^^50^^–^^53^^]^. • Planting for windbreaks/shelterbelts, wildlife habitat, soil erosion control facilitated^[^^54^^–^^58^^]^.
Eradication	Misaligned	Spread is encouraged and facilitated. Eradication only occurs at small scales within heavily-invaded landscapes.	• Planting for windbreaks/shelterbelts, wildlife habitat, soil erosion control facilitated^[^^50^^–^^58^^]^. • Management is not implemented until cover reaches 5–20%^[^^59^^,^ ^60^^,^ ^61^^]^. • Eradication implemented for small patches within eastern redcedar woodlands^[^^62^^–^^64^^]^. • Landowners encouraged to remove eastern redcedar by themselves at less than 5% cover^[^^56^^]^.
Control	Mostly aligned	Removal encouraged and facilitated, but control actions are typically implemented at local-scales. Planting is still facilitated.	• Planting for windbreaks/shelterbelts, wildlife habitat, soil erosion control facilitated^[^^50^^–^^58^^]^. • Cost-share for control available^[^^65^^,^ ^66^^]^.* • Control suggested before 70% cover is reached^[^^60^^,^ ^61^^]^.* • On average, management focuses on 50 acre land parcels^[^^31^^]^. • Mature crop trees must remain after cost-share removal^[^^65^^]^.
Long-term Management	Misaligned	Removal facilitated at local-scales to protect resources of importance, but more resources are allocated than theory supports. Planting is still facilitated.	• Planting for windbreaks/shelterbelts, wildlife habitat, soil erosion control facilitated^[^^50^^–^^58^^].^ • Large-scale management is considered economically infeasible^[^^60^^,^ ^61^^]^.* • Eastern redcedar removed to protect isolated grassland and livestock forage resources^[^^62^^–^^66^^]^.* • Financial incentive programs for eastern redcedar removal provide the greatest payments for the most heavily-invaded parcels^[^^59^^,^ ^65^^,^ ^66^^]^.

We found no evidence that the density-impact model was being used to guide practices and behaviors at the ecoregion extent. All ecoregions are managed as if they are in the control stage of the invasion process. However, Cherry County Wetlands falls within the prevention stage at the ecoregion extent (Table 1). The Central Loess Hills are at the eradication stage (Table 1), and the Loess Canyons were at levels of abundance consistent with the control stage (Tables 1 and 2). In summary, policies were implemented the same statewide, irrespective of the stage of invasion within each ecoregion (Table 2).

**Fig 7:**
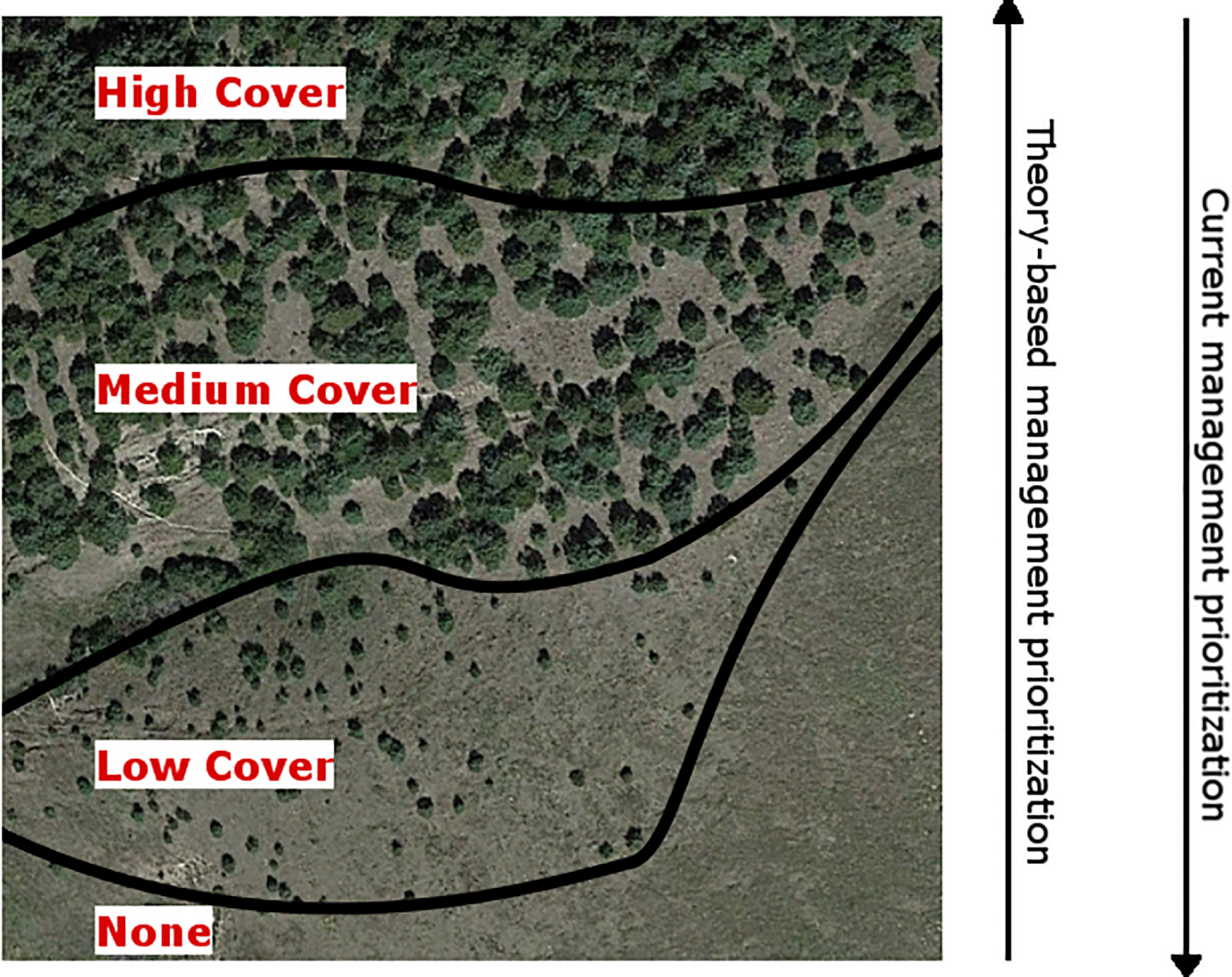
Agency policy prioritization for managing eastern redcedar invasions. Current natural resources agency prioritization (shown as downward-pointing triangle, where greater width indicates greater prioritization) is based on eastern redcedar density and spatial pattern of abundance, but this directly counters theoretical foundations that guide non-native species invasions (shown as upward-pointing triangle, where greater width indicates greater prioritization). The aerial image is from Google Earth.

For individual land parcels, the average spatial extent at which financial incentives programs are typically delivered (20-ha), policies failed to match the density-impact invasion curve, except at the control stage (Table 2; Fig 6). Policy-guided prevention of incipient invasions were not prioritized on individual parcels of land in any ecoregion (Fig 6). Advocating planting of eastern redcedar was more evident, followed by control on the same given 20.2 ha parcel (e.g., establishing a windbreak and then removing invasions resulting from the windbreak) [67]. For the eradication stage, text within one policy (Natural Resources Conservation Service) [61] that encouraged treatment at relatively low density levels, but conservation expenditures for preventative treatments received little to no actual financial support/enrollment and incentives for acting at the control stage were the majority [31]. At the control stage, policy was relatively consistent with the density-impact model (Table 2; Fig 6). However, we also found a control stage policy (from the Nebraska Forest Service) that states financial incentives or cost-share “CANNOT [emphasis theirs] be used to remove all tree vegetation from a site. A forest stand with [eastern red]cedar crop trees must remain after management” [65]. In areas where the stage of invasion varies greatly, such as the Loess Canyons and Central Loess Hills, the invasion model indicates that resources are typically best prioritized when invasive species are absent or in low abundance, and yet financial incentive programs prioritized funds in areas with greatest abundance (Fig 7; Table 2). Prevention in areas without eastern redcedar were given the least incentives (Fig 7). As an example, the Natural Resources Conservation Service’s Environmental Quality Incentives Program in 2018 will provide $196.48 per acre to help accomplish eastern redcedar removal in areas with high cover, $95.48 per acre for moderate cover, $37.50 per acre for low cover, and $0 for when eastern redcedar is absent [59]. Policy delineating control from long-term management was not obvious, in general, but one policy did indicate that management actions beyond a 70% cover threshold may be economically infeasible (Natural Resources Conservation Service) [61].

## Discussion

We demonstrate that eastern redcedar management policy is mired in a doublethink mentality between historical management strategies that assume eastern redcedar is in equilibrium with its environment, and therefore, facilitate its spread, while simultaneously managing eastern redcedar as a native invader outside of equilibrium during late invasion stages [21, 34, 68]. Stalling management until later invasion stages means that policy effectively tries to halt eastern redcedar spread at its most rapid rate. Unless the mismatch between science and policy is resolved, there is no evidence that the current management approach in Nebraska can stop grasslands from transitioning to eastern redcedar woodlands. Instead, both theory in invasion ecology and studies from other regions in the Great Plains on this native invader provide clear evidence for large-scale grass-to-woody transitions with profound impacts to ecosystem services (e.g., loss of education funding, livestock forage, and grassland ecosystems, increased pollen allergies, etc.) [9, 15, 30, 69].

Species invasions threaten ecosystem services at a range of scales from individual stakeholder land parcels to entire ecoregions, but policy and management actions often focus only on a single spatiotemporal scale or operate without consideration of the problems of scale in ecology [8, 16]. For instance, in Nebraska, although inter-agency management strategies (but not policies) are crafted at the extent of individual ecoregions [42], the Natural Resources Conservation Service typically administers management piecemeal on an individual landowner basis–50 acre (20.2 ha) parcels on average [31, 48]. This has led to local success in control and asset protection but has not affected the larger ecoregion or biome-scale transformation caused by eastern redcedar’s spread [9, 21]. Similarly, eastern redcedar management policies are not consistent among natural resource agencies and instead reflect a localized decision-making process and value-oriented disparities (e.g., wildlife habitat and windbreak plantings versus grassland restoration-motivated removal) that inhibit consistent, effective policy implementation.

Scale mismatches also reduce the potential for policy to meet sustainability targets. Information from one spatial extent can prevent policy change or necessary investments when information at another spatial scale warns of impending change within the socio-ecological system. This often results from misconceptions about the temporal scaling of invasion relative to the social constraints that limit the spatial extent of management interventions [24]. As a result, invasions can temporarily go undetected or be ignored, despite clear scientific knowledge showing temporal lags between introduction and rapid population growth [5, 12, 70–74]. This study provides a key example of scale mismatches in policy. For example, in the Cherry County Wetlands, the apparent lack of eastern redcedar spread at the ecoregion extent (percentage of ecoregion with juniper woodland = 0.02%) has been cited as an indication that eastern redcedar will never pose a threat in that ecoregion and can therefore be planted indiscriminately [15]. Counter to this logic, we demonstrate incipient invasions in the Cherry County Wetlands at the level of individual land parcels. This indicates that using a finer spatial resolution should be used to guide changes in program implementation would be more effective and science-supported, particularly given the economic costs of failing to act at the early stages of the invasion process. Understanding the scale to operationalize information and justify policy decisions that would therefore avoid tendencies to misidentify risks within an ecoregion. This is particularly important to the livelihoods for citizens within the Cherry County Wetlands, which is located in the Sandhills ecoregion—one of the largest intact grassland remaining in the USA, where the economy relies heavily on grasslands for livestock production.

To succeed at preventing or mitigating damage from invasive species, theory and literature emphasize the importance of matching management actions with the scale of invasion [4, 75, 76]. Policies and management actions that prevent or eradicate invasions in their early stages clearly match theory on the spatiotemporal scale of invasions, and consequently, are orders of magnitude more cost-effective than waiting for studies to find the most refined methods for combatting invasions [15, 16, 69]. For example, conducting a prescribed burn to eradicate eastern redcedar from grasslands capable of carrying fire can potentially have negligible costs, whereas the Nebraska Natural Resources Conservation Service’s policy indicates that lands with >70% eastern redcedar cover are economically infeasible to manage [31, 61]. Despite this, one example of doublethink is that policy from one agency provides tax-supported payment programs for eastern redcedar control that increase reimbursements with increasing eastern redcedar cover [77], while policy from another agency insists that some mature, seed-bearing eastern redcedar remain after control treatments to continue to facilitate their spread [65]. Because eastern redcedar does not resprout after cutting and high intensity burn treatments have been shown to cause 100% mortality even in mature trees [36], management at any invasion stage could potentially lead to eradication from locations where eastern redcedar is unlikely to have been present historically. However, fire ban policies, strict liability for fire damage, and lack of private citizen knowledge of safe prescribed burning techniques precludes high intensity fires for late-stage management in many cases [9, 24].

### Policy implications

Our results highlight the need for scientists, policy makers, and ecosystem managers to move past ideologies governing the classification of native versus non-native invaders and toward a new framework that takes into account the scale of invasions, the uniqueness of native species invasions in policy creation, and their impacts on ecosystem services. Doublethink policy approaches that that simultaneously acknowledge the spread of native invaders as problematic while facilitating that spread, as in the case of eastern redcedar in the Great Plains, can result in just as deleterious consequences as those associated with the spread of non-native invaders [5, 24]. Scientists could further advance the scientific basis of management for the native invader eastern redcedar, and others, by adapting additional existing models originally designed for non-native species which include their transport, distribution, scale issues, and colonization of new areas. By continuing to match and extend policy with current scientifically-based models, natural resource agencies can dispel doublethink in their policies and increase chances of success in invasive species management.

Federal policy directives governing invasive species management can be used as a path forward for local-to-regional policy revisions. Federal policies for invasive species have built-in controls meant to avoid doublethink (Fig 2). Agencies are not authorized to fund or conduct actions that cause or promote the introduction or spread of invasive species, unless public declarations have been made that the benefits of such actions clearly outweigh potential social and environmental damages [78]. Waiting to initiate management and/or to cease intentional introductions until it becomes a discernable socio-ecological problem represents a classical challenge in invasive species management that the executive mandate was meant to resolve (Fig 3). Federal policy also fosters transparency by requiring agencies to publicly declare that the benefits of promoting an invasive species clearly outweigh the potential for socioeconomic and environmental damages. For eastern redcedar invasions, policies make the equilibrium-based assumption that management can freeze eastern redcedar abundance within a desirable range that allows the benefits of planting while also minimizing or avoiding damages. However, literature and past invasive species management failures warn that these doublethink policies lack empirical support and are not science-based. The evidence instead indicates that such narratives over-promise benefits to society while also underplaying risks to the sustainability of ecosystem services into the future. In the case of eastern redcedar invasions, the risks to ecosystem services have been well-documented, but the local benefits (e.g., individual windbreak plantings) have not been shown to outweigh the economic losses associated with biome-level regime shifts across the Great Plains.

Understanding the risks of these equilibrial policy assumptions will allow policy-makers and natural resource agencies to craft ecologically-informed policies that question deeply-rooted practices known to erode resilience in socio-ecological systems. In reflexive law, the principle of information disclosure has been useful for providing the ability to shift away from past governance ideologies and toward more resilience-based governance structures when attempting to justify current recommendations and technical guidance [79]. This is the basis for federal disclosure of non-native invasions, and our study highlights the need to disclose such information for native invaders like eastern redcedar. We demonstrate an example of invasive species management policy that essentially communicates that the benefits of establishing eastern redcedar trees clearly outweighs the potential consequences (i.e., by promoting eastern redcedar regardless of invasion stage). This clear example of doublethink counters the current scientific consensus and prevents the adaptation of existing policy to include preventative actions under the authority of natural resource professionals.
